# Case report: a case report of neoadjuvant mFOLFIRINOX leading to a partial pathologic response in pancreatic adenosquamous carcinoma

**DOI:** 10.1093/jscr/rjae345

**Published:** 2024-05-26

**Authors:** Deepak Dev Vivekanandan, Hardeep Singh, Nelson Andrew Royall

**Affiliations:** Department of Surgery, Northeast Georgia Medical Center, 743 Spring Street NE, Gainesville, GA 30501, United States; Graduate Medical Education, Research Team, Northeast Georgia Medical Center, 743 Spring Street NE, Gainesville, GA 30501, United States; Department of Surgery, Northeast Georgia Medical Center, 743 Spring Street NE, Gainesville, GA 30501, United States

**Keywords:** pancreatic adenosquamous carcinoma, neoadjuvant mFOLFIRINOX, Whipple procedure, SMA invasion, SMV invasion, partial response

## Abstract

A female in her 60s with vague abdominal symptoms was found to have a pancreatic mass in her CT scan. A core needle biopsy done endoscopically demonstrated a poorly differentiated adenocarcinoma. The patient completed nine cycles of neoadjuvant systemic mFOLFIRINOX. Repeat staging demonstrated a partial radiographic response. She underwent an open pylorus-preserving pancreatoduodenectomy with segmental superior mesenteric vein resection with primary reconstruction (ISGPS Type 3). The final pathology demonstrated a poorly differentiated adenosquamous carcinoma, R1 margin status. The case report demonstrates the effect of mFOLFIRINOX on pancreatic adenosquamous (PASC) carcinoma with a review of the microscopic pictures following the neoadjuvant therapy. It can be postulated that glandular component being the major component in a PASC has a good response to mFOLFIRINOX like that seen in pancreatic ductal adenocarcinoma with some presumed effect on the squamous component as well. From the above case report, we are proposing that mFOLFIRINOX can be an effective chemotherapy regime in the management of PASC.

## Introduction

Pancreatic adenosquamous (PASC) carcinoma is a rare histologic type of pancreatic malignancy, which represents 1–4% of pancreatic malignancies [1–3]. PASC is associated with worse overall survival (11.8 months) and is diagnosed at more advanced stages compared with pancreatic ductal adenocarcinoma (PDAC) [1, 3]. Histologically, PASC has a combination of glandular and squamous components, requiring tailoring the chemotherapy regime to be effective against both components.

Clinical trials have demonstrated neoadjuvant systemic chemotherapy, involving either mFOLFIRINOX or gemcitabine and nab-paclitaxel, as the standard of care for borderline resectable and locally advanced PDAC [4–6]. Due to the rarity of PASC, there is no clear evidence for the utilization of a specific neoadjuvant regimen for borderline resectable and locally advanced PASC. This case describes a patient found to have PASC who completed neoadjuvant mFOLFIRINOX with a partial pathologic response.

## Case report

**Figure 1:**
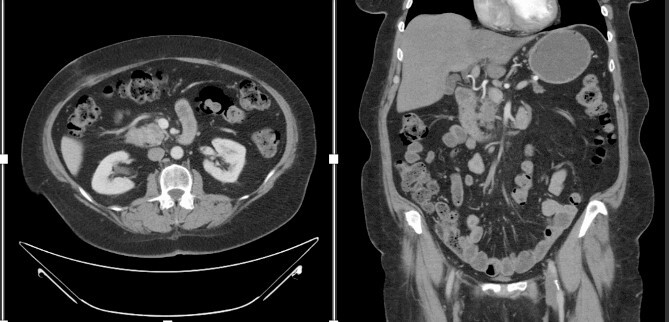
CT abdomen and pelvis with IV contrast with axial and coronal views demonstrating the Pancreatic mass.

A female in her 60s presented with a 6-month history of progressive abdominal cramping, acholic stools, early satiety, post-prandial loose stools and flatulence. She reported a history of newly diagnosed diabetes mellitus with progressively difficult-to-control hyperglycemia requiring initiation of GLP-1 agonist therapy within the preceding year. She was a former cigarette smoker with 15 pack years’ history and no reported other substance use history. Basic hematological test was normal. Colonoscopy done showed no significant findings. A CT abdomen and pelvis with contrast identified a hypodense 1.5-cm solid mass in the pancreatic head with abutment of the superior mesenteric vein without arterial abutment or regional lymphadenopathy (Fig. 1); proximal main pancreatic duct dilatation and pancreatic parenchymal atrophy in the neck, body and tail. An endoscopic ultrasound identified a 2.8-cm hypoechoic partially circumscribed 1.5-cm mass in the pancreatic head. Core biopsy demonstrated poorly differentiated adenocarcinoma (PanKeratin, S100p, DOG-1, CK-7 positive; Ki67 20–30%). A staging CT chest and pancreatic protocol demonstrated an interval increase of the solid pancreatic head mass to 2.5 cm without other abnormalities (Fig. 1). Baseline serum Cancer Antigen 19-9 was non-elevated (3.02 U/mL). She was staged as a cT2N0M0 (Stage IB) pancreatic adenocarcinoma prior to therapy. Germline genetic testing demonstrated variants in KRAS, CDKN2A and TP53 with variant allele fraction of 25, 24.85 and 20.3%, respectively. Microsatellite instability was stable with a tumor mutation burden of 3.2 m/MB. She was initiated on pancreatic enzyme replacement therapy with resolution of her presenting symptoms and glycemic control was achieved with GLP-1 agonist therapy. The patient was referred for Genetic counselling.

She completed nine cycles of mFOLFIRINOX systemic therapy, requiring a 50% irinotecan dose reduction at cycle 3 and a 75% reduction at cycle 4 due to neurocognitive side-effects. She discontinued systemic therapy after cycle 9 due to progressive chemotherapy-associated steatohepatitis and fatigue. Repeat staging imaging demonstrated a RECIST 1.1 radiographically stable disease (12% reduction in the primary tumor diameter).

**Figure 2:**
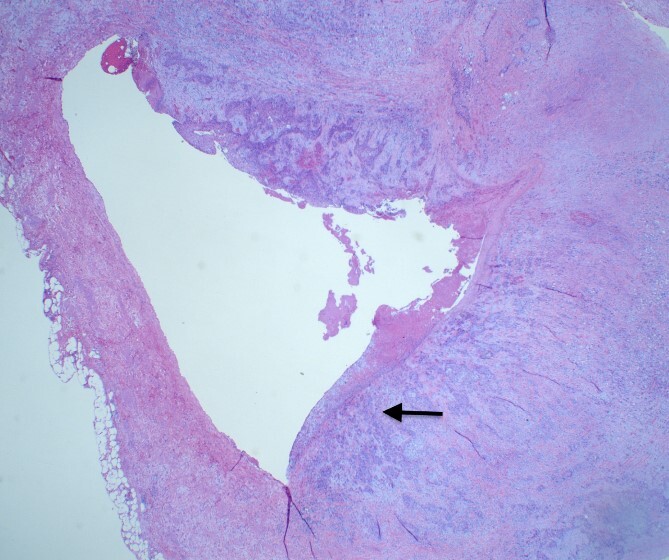
H&E slide demonstrating SMV invasion

**Figure 3:**
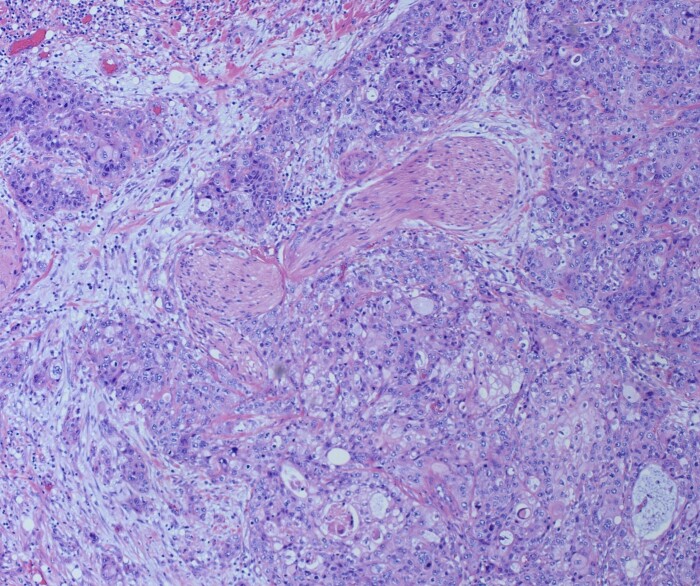
H&E Slide demonstrating perineural invasion

**Figure 4:**
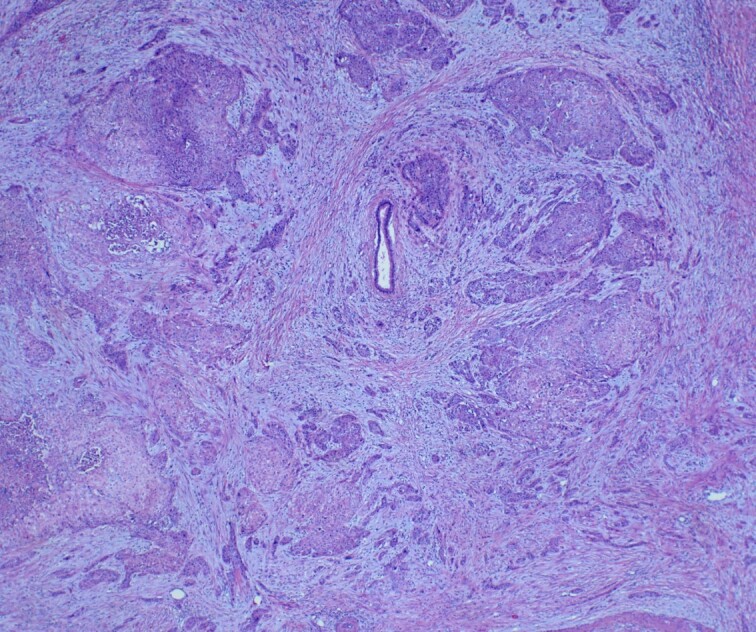
Adenosqumous carcinoma with both components (adeno and squamous) A19 Cytokeratin 7 immunostain, highlighting the adenocarcinoma, and p40 immunostain, highlighting the squamous carcinoma component

**Figure 5:**
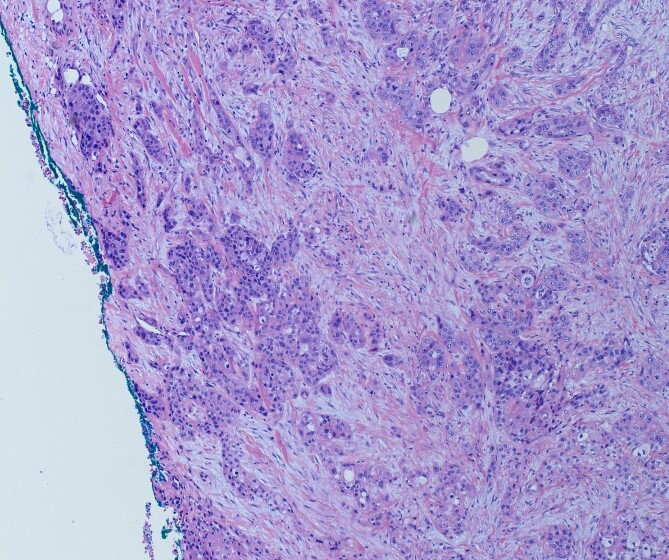
H&E slide demonstrating superior mesenteric artery invasion

She underwent an open pylorus-preserving pancreatoduodenectomy with segmental superior mesenteric vein resection with primary reconstruction (ISGPS Type 3) without complication. Final pathology demonstrated a 3.6-cm poorly differentiated adenosquamous carcinoma with SMV invasion into the intima (Fig. 2), 1 of 24 regional lymph nodes with metastatic carcinoma, lymphovascular and perineural invasion (Fig. 3), and an 85% squamous differentiation (Fig. 4). There was a partial pathologic response (College of American Pathologist Grade 2) within the primary tumor and involved lymph node tumor [7]. There was noted to be tumor extension within 1 mm of the superior mesenteric artery margin (Fig. 5) (R1 status) with negative additional margins. She recovered without complications and was discharged home on the fourth post-operative day. Repeat staging CT imaging demonstrated no evident disease. She was initiated on adjuvant systemic therapy.

## Discussion

Even though an arbitrary cutoff has been disputed, PASC historically was defined by the presence of at least 30% of malignant squamous component in the background of adenocarcinoma [8]. Core needle biopsy is susceptible to falsely identifying PDAC in the setting of PASC due to the limited evaluation of the squamous component within the tumor. Serum CA19-9 can be elevated in patients with PASC and may be used as a biomarker of treatment response, although patients without the Lewis antigen are similarly unable to secrete CA19-9, limiting its broader application [9].

No treatment guidelines exist for PASC due to the low incidence [3]. Overall survival for PASC is shorter compared to PDAC on a stage-for-stage basis [10, 11]. Surgical resection remains the mainstay of treatment, with a consistent improvement in overall survival in the non-metastatic setting [9, 11] Adjuvant systemic chemotherapy has been associated with improved overall survival in small retrospective case series, with predominantly 5-FU or Gemcitabine-based regimens [12–14]. Case reports have documented complete response of PASC to Gemcitabine and platinum-based chemotherapy as well as gemcitabine, nab-paclitaxel and pembrolizumab [13, 15].

Neoadjuvant systemic therapy with mFOLFIRINOX has been shown to have an improved overall survival(OS) for borderline resectable PDAC based on the Alliance A021501 trial with a 66.4% 18-month OS [5]. This patient demonstrated a 12% reduction in the primary tumor diameter radiographically following nine cycles of mFOLFIRINOX. The patient demonstrated a pathologic partial response (CAP Grade 2) following the administration of mFOLFIRINOX in a similar course to that of the A021501 trial, which involved eight cycles followed by surgery. The factors contributing to the response in this patient are likely related to the dominant histologic features being similar to that of PDAC. This patient was found to have a similar somatic mutational status as that of PDAC with KRAS, TP53 and CDKN2A mutations [16]. Similarly, mFOLFIRINOX likely generates a response in the squamous component of the tumor as squamous carcinomas are generally known to be platinum-chemotherapy sensitive [17].

## Conclusion

- The patient demonstrated a partial pathologic response to mFOLFIRINOX in the neoadjuvant setting.
- The finding of a similar somatic mutational status generates the hypothesis that this pathologic response is related to a similar pathway as that of PDAC.
- For patients with PASC, consideration of neoadjuvant mFOLFIRINOX may be a reasonable treatment strategy based on this case report.
